# Routine laboratory surveillance of antimicrobial resistance in community-acquired urinary tract infections adequately informs prescribing policy in England

**DOI:** 10.1093/jacamr/dlaa022

**Published:** 2020-05-27

**Authors:** Vicky Watts, Benjamin Brown, Maria Ahmed, André Charlett, Carolyn Chew-Graham, Paul Cleary, Valerie Decraene, Kirsty Dodgson, Ryan George, Susan Hopkins, Aneez Esmail, William Welfare

**Affiliations:** 1 Field Service North West, National Infection Service, Public Health England, Liverpool, UK; 2 Centre for Primary Care, Division of Population Health, School of Health Sciences, Faculty of Biology, Medicine and Health, University of Manchester, Manchester, UK; 3 Centre for Health Informatics, Division of Informatics, Imaging and Data Science, School of Health Sciences, Faculty of Biology, Medicine and Health, University of Manchester, Manchester, UK; 4 Manchester Medical, Moss Side Health Centre, Manchester, UK; 5 NIHR Clinical Research Network: Greater Manchester, Manchester, UK; 6 Statistics Unit, Data and Analytical Sciences, National Infection Service, Public Health England, London, UK; 7 School of Primary, Community and Social Care, Keele University, Keele, Newcastle-under-Lyme, UK; 8 Manchester University NHS Foundation Trust, Manchester, UK; 9 HCAI & AMR Division, National Infection Service, Public Health England, London, UK; 10 NIHR School for Primary Care Research, Manchester Academic Health Science Centre, University of Manchester, Manchester, UK; 11 Health Protection Team, Public Health England North West, Manchester, UK; 12 Division of Population Health, Health Services Research and Primary Care, School of Health Sciences, Faculty of Biology, Medicine and Health, University of Manchester, Manchester, UK

## Abstract

**Objectives:**

To assess whether resistance estimates obtained from sentinel surveillance for antimicrobial resistance (AMR) in community-acquired urinary tract infections (UTIs) differ from routinely collected laboratory community UTI data.

**Methods:**

All patients aged ≥18* *years presenting to four sentinel general practices with a suspected UTI, from 13 November 2017 to 12 February 2018, were asked to provide urine specimens for culture and susceptibility. Specimens were processed at the local diagnostic laboratory. Antibiotic susceptibility testing was conducted using automated methods. We calculated the proportion of *Escherichia coli* isolates that were non-susceptible (according to contemporaneous EUCAST guidelines) to trimethoprim, nitrofurantoin, cefalexin, ciprofloxacin and amoxicillin/clavulanic acid, overall and by age group and sex, and compared this with routine estimates.

**Results:**

Sentinel practices submitted 740 eligible specimens. The specimen submission rate had increased by 28 specimens per 1000 population per year (95% CI 21–35). Uropathogens were isolated from 23% (169/740) of specimens; 67% were *E. coli* (113/169)*.* Non-susceptibility of *E. coli* to trimethoprim was 28.2% (95% CI 20.2–37.7) on sentinel surveillance (33.4%; 95% CI 29.5–37.6 on routine data) and to nitrofurantoin was 0.9% (95% CI 0–5.7) (1.5%; 95% CI 0.7–3.0 on routine data).

**Conclusions:**

Routine laboratory data resulted in a small overestimation in resistance (although the difference was not statistically significant) and our findings suggest that it provides an adequate estimate of non-susceptibility to key antimicrobials in community-acquired UTIs in England. This study does not support the need for ongoing local sentinel surveillance.

## Introduction

Urinary tract infections (UTIs) are among the most common infections managed in primary care,^1^ estimated to affect 50% of women during their lifetime.^2^ UTI is one of the diagnoses for which antibiotics are most frequently prescribed.^3^ Antimicrobial resistance (AMR) is increasing among uropathogens.^4–7^ Resistant strains of *Escherichia coli*, the pathogen most frequently associated with community-acquired UTIs, are driving an increased incidence of *E. coli* bacteraemia.^8^

Guidelines for the management of uncomplicated UTIs in primary care in England recommend urine culture from patients with clinical treatment failure, frequent or recurrent UTIs or a likelihood of resistant infection.^1^^,^^5^^,^^9^^,^^10^ Otherwise, empirical treatment is recommended as uncomplicated infections are generally caused by a narrow range of pathogens that historically have had a predictable antibiotic susceptibility profile.^10^^,^^11^ Guidelines have recently recommended nitrofurantoin as the first-line treatment, in preference to trimethoprim, as the prevalence of trimethoprim resistance exceeds 20% in adult patients.^10–13^

Prescribing guidance is informed by routine laboratory surveillance. Approximately 98% of hospital microbiology laboratories in England voluntarily report routine antimicrobial susceptibility testing (AST) results, with patient demographic information, to the PHE national laboratory surveillance system [Second Generation Surveillance System (SGSS)].^1^^,^^12^ This system has the potential to overestimate resistance as it is based upon isolates submitted for diagnostic testing and is thus more likely to have resistant infections. This may result in guidelines being amended unnecessarily, potentially leading to the higher use of second-line agents and broad-spectrum antibiotics, further driving resistance.^9^^,^^14^^,^^15^

Sentinel surveillance, which would base resistance estimates on AST data from specimens collected from a wider range of patients, was a key recommendation of the English Surveillance Programme for Antimicrobial Utilisation and Resistance (ESPAUR) 2017 and has the potential to provide a less biased estimate of resistance.^5^^,^^9^ We established a pilot sentinel surveillance system for AMR in community-acquired UTIs in Greater Manchester to determine whether current routine surveillance data for *E. coli* accurately estimate resistance.

## Materials and methods

### Design

We conducted a prospective pilot surveillance study of AMR among community-acquired UTIs in Greater Manchester.

### Study population and definitions

Clinicians at sentinel practices were asked to request urine specimens from all registered patients aged ≥18 years presenting with symptoms suggestive of a UTI between 13 November 2017 and 12 February 2018 (when planned sample size was reached). The study was designed to follow routine clinical practice as closely as possible. Diagnosis was therefore based on clinical judgement; we did not define the diagnostic criteria. A confirmed UTI was defined as the demonstration of significant bacteriuria by culture: for women a count of ≥10^5^ cfu/L of a single isolate and for men a count of ≥10^6^ cfu/L of a pure or predominant organism.^16^

The following specimens were excluded:

- a repeat urine specimen submitted during the pilot (we included the first urine specimen submitted only)
- a specimen submitted as an antenatal screening sample
- a specimen submitted due to previous treatment failure for a UTI.

GP practices provided this information for the latter two electronically at the time of specimen submission to the laboratory. Patients were managed according to routine clinical practice.

### Sampling methods

A convenience sample of four general practices was selected to participate, based on:

- use of the Manchester University Foundation Trust (MFT) central laboratory site for the routine processing of urine samples
- the number of samples they submitted to this laboratory in 2016 (practices submitting a higher number of samples were selected to reduce the number of practices required and the duration of the study to meet the required sample size)
- willingness to participate in the study.

The study was powered to estimate non-susceptibility of *E. coli* isolates to key antimicrobials from sentinel surveillance data. Given our hypothesis that routine data overestimate resistance, we based our sample size on 10% non-susceptibility of *E. coli* to trimethoprim, with 5% precision, giving a required sample size of 139 positive samples. Twenty-five percent of community urine specimens submitted to the MFT laboratory in 2016 were positive for a uropathogen; as we intended to request specimens from a wider range of patients, we estimated that positivity would reduce to 12.5% and therefore that we would require 1112 specimens to obtain the required sample size.

### Data collection

#### Laboratory analysis

Specimens were transported to the laboratory and analysed at the MFT central laboratory site according to routine procedures: specimens were processed using the Sysmex UF1000i Urine analyser (Sysmex, Hyogo, Japan) or via manual microscopy, where indicated. Culture was performed on Brilliance UTI Clarity chromogenic agar (Oxoid Ltd., Basingstoke, UK) using the PREVI Isola (bioMérieux, Marcy l’Étoile, France). Species identification was based on chromogenic agar only except for non-*E. coli* coliforms, where identification of isolates was performed on the MALDI-TOF Microflex LT System GGA004213 (Bruker UK Ltd, Coventry, UK). Antimicrobial susceptibility was determined using the VITEK 2 XL analyser (bioMérieux, Marcy l’Étoile, France) (EUCAST breakpoints).^17^ Disc diffusion methods using BSAC breakpoints were employed to determine sensitivities for *Enterococcus* spp. and possible ESBL-producing isolates to allow targeted testing of relevant antibiotics.

#### Data sources

Demographic and laboratory information for the specimens submitted from sentinel practices and from all NHS Manchester Clinical Commissioning Group (CCG) practices (termed ‘Manchester practices’ from this point) that submitted specimens to the MFT laboratory between 13 November 2017 and 12 February 2018 were obtained from the laboratory information management system (these data are also submitted to the national surveillance system, SGSS). Details of specimens submitted from all NHS Manchester CCG practices over the same period were obtained to represent routine data for comparison with sentinel resistance estimates. The same data fields were also extracted for specimens submitted from the four sentinel practices for the same period of the previous year (13 November 2016 to 12 February 2017) to allow comparison of the age–sex distribution of patients who submitted samples.

### Data analysis

#### Calculating non-susceptibility

We calculated the proportion of *E. coli* isolates tested for susceptibility from sentinel and Manchester practices that were non-susceptible to trimethoprim, nitrofurantoin, cefalexin, ciprofloxacin or amoxicillin/clavulanic acid, overall and by age group and sex. In accordance with contemporaneous EUCAST guidelines at the time of the study, ‘resistant’ and ‘intermediate’ isolates were combined and reported as ‘non-susceptible’.^17^

#### Representativeness

To establish whether specimens were submitted from a wider range of patients, we divided the number of patients who submitted a urine specimen at each practice by the number of patients coded as having a suspected UTI on the GP patient management system. We also calculated the rate of specimen submission per 1000 population for each GP practice in NHS Manchester CCG that submitted specimens to the MFT laboratory (including sentinel practices) during 2015, 2016 and the pilot:

$$\frac{\text{Number of community urine specimens submitted from patients aged }\geq \text{18 years registered at GP practice A, 2015}}{\text{Number of patients aged}\geq \text{18 years registered at GP practice A, 2015 }}\text{×1000}$$

The number of patients registered at each GP practice per year was obtained from NHS Digital (https://digital.nhs.uk/data-and-information/publications/statistical/patients-registered-at-a-gp-practice). The July GP list size was used for 2015 and 2016 and the December GP list size was used for 2017 for the pilot in order to represent a midpoint estimate for each period. The number of community urine specimens submitted during 2015 and 2016 was obtained from the MFT laboratory. The distribution of specimen submission rate by each NHS Manchester CCG GP practice was plotted for 2015, 2016 and for the pilot. The increase in specimen submission rate between the pilot and 2015 (practices B, C, D) and 2016 (practice A; numerator data were not available for practice A in 2015) was calculated. (There was a sharp reduction in the number of specimens submitted from NHS Manchester CCG practices in 2016, therefore 2015 was used as a comparison.) We compared the distribution of the number of specimens submitted by age–sex category at sentinel practices during the pilot and during 2015 (practices B, C and D) and 2016 (practice A) to determine whether any increase in specimen submission was focused on a particular age–sex category.

We compared the age–sex distribution of the patients who submitted urine specimens from sentinel practices during the pilot with all Manchester practices over the same period and with sentinel practices during the same period of the previous year. To further assess the representativeness of the system we compared the practice list size and age distribution of registered patients (using information from the PHE fingertips general practice profile, https://fingertips.phe.org.uk/profile/general-practice) with the NHS Manchester CCG average and the ethnicity profile with 2011 Census data for the Manchester local authority area.

### Ethics

PHE Research Ethics and Governance Group approval was granted for this study. Patients were managed according to routine clinical practice and therefore consent outside normal clinical practice was not required.

## Results

**Figure 1.**
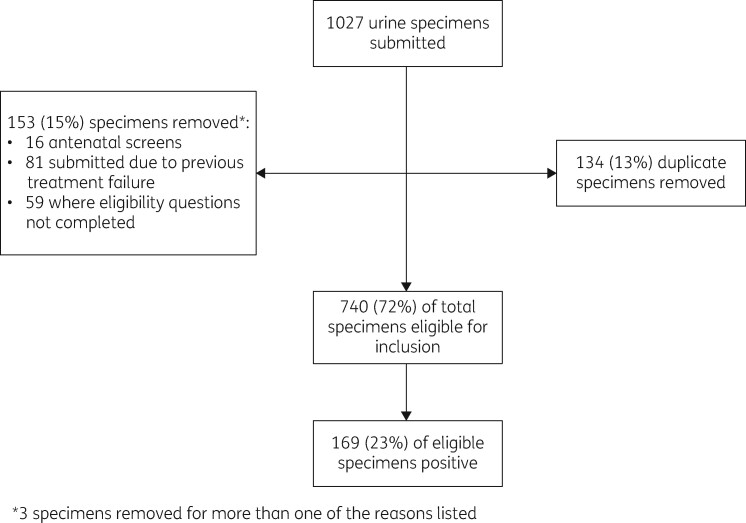
Urine specimens submitted by sentinel practices during a pilot surveillance study for AMR among community-acquired UTIs, 13 November 2017 to 12 February 2018.

Sentinel practices submitted 740 eligible specimens during the pilot. Figure 1 summarizes the specimens removed at each stage. Twenty-three percent (169/740) were positive for a uropathogen; *E. coli* was isolated from 67% (113/169) of positive specimens. *Klebsiella pneumoniae* was the next most commonly isolated organism (9%) followed by *Citrobacter koseri* (5%), *Enterococcus* spp. (5%) and *Staphylococcus saprophyticus* (3.5%). Twenty-three percent (818/3626) of specimens captured by routine data were positive for UTI. The proportion of specimens submitted in each age group that were positive for a uropathogen was not significantly different between sentinel and routine data (*χ*^2^ = 2.50; df = 2; *P *=* *0.29).

### Non-susceptibility estimates

Crude sentinel surveillance estimates of *E. coli* non-susceptibility to the antimicrobials tested were between 0.6% and 6.3% lower compared with routine surveillance; these differences were not statistically significant (although the study was not powered to be able to detect this) (Table 1). Twenty-eight percent (95% CI 20.2–37.7) of *E. coli* isolates from sentinel practices were not susceptible to trimethoprim, compared with 33% (95% CI 29.5–37.6) in routine data. Non-susceptibility to nitrofurantoin was 0.9% (95% CI 0–5.7) on sentinel surveillance, compared with 1.5% (95% CI 0.7–3.0) in routine data (Table 1).

**Table 1.** Proportion of *E. coli* isolates non-susceptible to antimicrobials tested by age and sex, sentinel and routine data, 13 November 2017 to 12 February 2018

Antibiotic	Sex	Age group, years	Sentinel data			Routine data			Difference in proportion non- susceptible (95% CI)
			tested	non- susceptible	percentage (95% CI)	tested	non- susceptible	percentage (95% CI)	
Trimethoprim									
	F	18–44	55	15	27.3 (16.5–41.2)	254	75	29.5 (24.1–35.6)	−2.2 (−16.4 to 11.9)
		45–64	24	7	29.2 (13.4–51.3)	108	44	40.7 (31.5–50.6)	−11.5 (−34.5 to 11.4)
		≥65	20	6	30.0 (12.8–54.3)	121	41	33.9 (25.7–43.1)	−3.9 (−28.6 to 20.8)
	M	18–44	1	0	0.0 (0.0–94.5)	16	5	31.3 (12.1–58.5)	−31.3 (−85.2 to 22.7)
		45–64	6	0	0.0 (0.0–48.3)	20	4	20.0 (6.6–44.3)	−20 (−48.4 to 8.4)
		≥65	4	3	75.0 (21.9–98.7)	22	12	54.6 (32.7–75.0)	20.4 (−41.6 to 82.5)
	total		110	31	28.2 (20.2–37.7)	542^a^	181	33.4 (29.5–37.6)	−5.2 (−15.1 to 4.6)
Nitrofurantoin									
	F	18–44	55	0	0.0 (0.0–8.1)	254	3	1.2 (0.3–3.7)	−1.2 (−3.6 to 1.3)
		45–64	24	0	0.0 (0.0–17.2)	108	0	0.0 (0.0–4.3)	0
		≥65	20	1	5.0 (0.3–27.0)	121	3	2.5 (0.6–7.6)	2.5 (−9.9 to 15.0)
	M	18–44	1	0	0.0 (0.0–94.5)	16	0	0.0 (0.0–24.1)	0
		45–64	6	0	0.0 (0.0–48.3)	20	1	5.0 (0.3–27.0)	−5.0 (−19.6 to 9.6)
		≥65	4	0	0.0 (0.0–60.4)	22	1	4.6 (0.24–24.9)	−4.6 (−17.8 to 8.7)
	total		110	1	0.9 (0.0–5.7)	542^a^	8	1.5 (0.7–3.0)	−0.6 (−3.2 to 2.0)
Amoxicillin/ clavulanic acid									
	F	18–44	55	19	34.6 (22.6–48.7)	254	104	40.9 (34.9–47.3)	−6.3 (−21.5 to 8.7)
		45–64	24	7	29.2 (13.4–51.3)	108	50	46.3 (36.7–56.1)	−17.1 (−40.1 to 5.9)
		≥65	20	9	45.0 (23.8–68.0)	121	55	45.5 (36.5–54.7)	−0.5 (−24.4 to 23.5)
	M	18–44	1	1	100.0 (5.5–100.0)	16	5	31.3 (12.1–58.5)	68.7 (−7.1 to 100)
		45–64	6	3	50.0 (18.8–81.2)	20	10	50.0 (29.9–70.1)	0 (−45.6 to 45.6)
		≥65	4	3	75.0 (21.9–98.7)	22	16	72.7 (49.6–88.4)	2.3 (−46.3 to 50.1)
	total		110	42	38.2 (29.2–48.0)	542^a^	241^a^	44.5 (40.2–48.8)	−6.3 (−16.8 to 4.3)
Ciprofloxacin									
	F	18–44	55	7	12.7 (5.7–25.1)	254	28	11.0 (7.57–15.69)	1.7 (−9.0 to 12.4)
		45–64	24	4	16.7 (5.5–38.2)	108	20	18.5 (11.94–27.39)	−1.82 (−20.3 to 16.6)
		≥65	20	2	10.0 (1.8–33.1)	121	23	19.0 (12.67–27.37)	−9.0 (−26.8 to 8.8)
	M	18–44	1	0	0.0 (0.0–94.5)	16	3	18.8 (5.0–46.3)	−18.8 (−56.6 to 19.1)
		45–64	6	0	0.0 (0.0–48.3)	20	4	20 (6.6–44.3)	−20 (−48.4 to 8.4)
		≥65	4	1	25.0 (1.3–78.1)	22	8	36.4 (18.0–59.2)	−11.4 (−69.7 to 47.0)
	total		110	14	12.7 (7.4–20.1)	542^a^	86	15.9 (12.9–19.3)	−3.2 (−10.6 to 4.4)
Cefalexin									
	F	18–44	55	4	7.3 (2.4–18.4)	254	19	7.5 (4.7–11.6)	−0.2 (−8.0 to 7.6)
		45–64	24	1	4.2 (0.2–23.1)	108	13	12.0 (6.8–20.1)	−7.8 (−20.5 to 4.8)
		≥65	20	2	10 (1.8–33.1)	121	20	16.5 (10.6–24.6)	−6.5 (−24.1 to 11.1)
	M	18–44	1	0	0 (0.0–94.5)	16	2	12.5 (2.2–39.6)	−12.5 (−41.2 to 16.2)
		45–64	6	1	16.7 (0.9–63.5)	20	3	15.0 (4.0–38.9)	1.7 (−33.7 to 37.0)
		≥65	4	0	0 (0.0–60.4)	22	7	31.8 (14.7–54.9)	−31.8 (−66.1 to 2.4)
	total		110	8	7.3 (3.4–14.3)	542^a^	65	12.0 (9.4–15.1)	−4.7 (−10.8 to 1.4)

F, female; M, male.

a Sex or age group unknown for some patients therefore age–sex denominators may not sum to total.

Routine data overestimated resistance to trimethoprim, cefalexin and amoxicillin/clavulanic acid to a greater extent in females aged 45–64 years and to ciprofloxacin and cefalexin in females aged ≥65 years compared with other age–sex categories; these differences were not statistically significant but the study was not powered to detect this (Table 1).

### Representativeness

**Figure 2.**
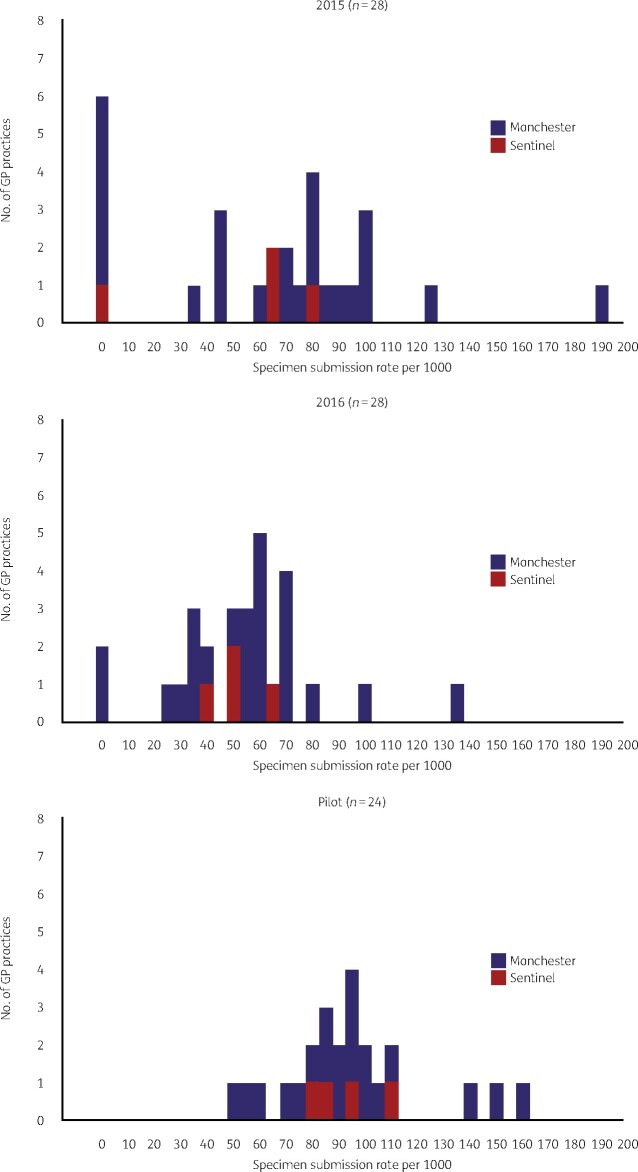
Distribution of urine specimen submission rate at NHS Manchester CCG GP practices using the MFT laboratory: 2015, 2016 and sentinel surveillance pilot.

Sentinel practices were not at the extremes of the distribution of specimen submission rate in 2015 and 2016 (Figure 2). Specimen submission rate increased by 28 specimens per 1000 population (95% CI 21–35) compared with 2015–16. The increase in specimen submission rate at sentinel practices was evenly distributed across all age–sex categories (female: *χ*^2^ = 1.24; df = 2; *P *=* *0.54; male: *χ*^2^ = 1.68; df = 2; *P *=* *0.43). The age distribution of patients who submitted samples from sentinel practices during the pilot was not significantly different from those captured by routine data (*χ*^2^ = 2.2; df = 2; *P *=* *0.30) during the same period or from those from the same practices during the same period of 2016–17 (*χ*^2^ = 5.0; df = 2; *P *=* *0.08).

From practices C and D, specimens were submitted from 89% and 98% of all patients coded as having a suspected UTI during the pilot (this measure was unavailable for practice B and invalid for practice A as more specimens were submitted than patients coded as having a suspected UTI (218 urine specimens submitted; 166 patients coded as having a UTI). There was variation in the practice and population catchment characteristics of sentinel practices compared with NHS Manchester CCG and the local authority average (Table S1, available as Supplementary data at *JAC-AMR* Online). Practice C, which contributed the most specimens, had a higher proportion of patients aged 15–44 years: 69% of registered patients compared with the NHS Manchester CCG average of 51%. Practices B, C and D (contributing 64% of specimens) had a higher proportion of patients of black and Asian ethnicity than the Manchester local authority average (Table S1).

## Discussion

### Summary of findings

Routine surveillance overestimated resistance in community-acquired UTIs to key antimicrobials by between 0.8% and 6.3%. Non-susceptibility of *E. coli* to trimethoprim on sentinel surveillance was estimated to be 28% (95% CI 20.2–37.7), compared with 33% (95% CI 29.5–37.6) based on routine data. For nitrofurantoin, the sentinel estimate of non-susceptibility was 0.9% (95% CI 0–5.7), compared with 1.5% (95% CI 0.7–3.0) based on the routine data. However, these small overall differences masked larger variation by age group and sex, with the greatest difference between sentinel and routine estimates occurring in female patients aged over 45 years for trimethoprim, cefalexin, ciprofloxacin and amoxicillin/clavulanic acid. Although these differences were not statistically different, this study was not powered to detect this and may be indicative of overestimation to a greater extent in these age groups.

### Implications for practice

Our findings suggest that routine data slightly overestimate resistance among community urinary pathogens to key antimicrobials in Manchester, not to an extent, however, that should influence selection of first-line antimicrobials and thus would not support a policy change regarding surveillance. This study suggests that routine data provide a reasonable estimate of non-susceptibility to key antimicrobials on which to base local prescribing recommendations and supports the recent change from trimethoprim to nitrofurantoin as the first-line treatment for uncomplicated UTIs in primary care in England.^13^ Nevertheless, this study indicates a true overestimation of resistance by routine data, which, although small, may result in the threshold for a change in antibiotic prescribing practice being reached earlier than necessary.

### Comparison with existing literature

Our findings are supported by a recent analysis in England that demonstrated similar resistance to trimethoprim and nitrofurantoin in *E. coli* urinary isolates from children <5 years when comparing data from SGSS and a prospective study.^12^ However, McNulty *et al.*^18^ demonstrated that routine data overestimated *E. coli* resistance to trimethoprim in adult females presenting to primary care in England with symptoms of uncomplicated UTI by 10%–13%. Two studies in Germany also demonstrated a 10%–11% overestimation of *E. coli* resistance to trimethoprim in adult women in primary care with uncomplicated UTI.^14^^,^^19^ They concluded that routine laboratory surveillance had limited value in informing empirical therapy for uncomplicated UTI, with Schmiemann *et al.*^19^ recommending sentinel surveillance to monitor resistance in primary care.^14^ We aimed to mirror routine practice as far as possible to maximize recruitment and did not collect any clinical or risk factor information about the patients so that practices did not have to recruit individual patients nor obtain individual patient consent. We were therefore unable to stratify resistance estimates by complicated and uncomplicated UTI nor specific risk factors for treatment failure such as indwelling urinary catheters, which may partly explain the smaller differences we found between sentinel and routine data. Only 23% of eligible urine specimens submitted were positive for a uropathogen. A systematic review reported positivity of 28%–67% for women presenting with uncomplicated UTI, where the same threshold for a positive culture of ≥10^5^ cfu/L was used.^20^ Increasing the specificity of our case definition through defining a UTI by the presence of two or more key symptoms would have increased the positivity.^18^ However, low recruitment is a recognized barrier to conducting studies in primary care;^18^ therefore we opted for a pragmatic case definition based on clinical judgement. The positivity rate in our study was reflected by routine data suggesting that qualitative work to determine the criteria for diagnosing UTIs in primary care in Greater Manchester may be of value to improve ascertainment and therefore reduce inappropriate prescribing. Further work is needed to determine the appropriate threshold to define a positive culture.

### Strengths and limitations

The key strengths of this study were that sentinel and routine specimens were processed at the same laboratory, and therefore resistance estimates were directly comparable, and by mirroring routine clinical practice we could provide a relatively rapid assessment of AMR in the community, collecting sufficient specimens within just 4 months.

However, our pragmatic approach introduced several limitations, including the specificity of the case definition and combined estimate of non-susceptibility for complicated and uncomplicated UTI discussed above. Recruitment of GP practices was not random and although resistance estimates were based on a less biased sample, variation in patient characteristics between populations under surveillance may explain some of the difference in non-susceptibility estimates. Sentinel practices were representative of other NHS Manchester CCG practices in terms of their specimen submission rate before the pilot. Had the specimen submission rate been higher at sentinel practices compared with other GP practices prior to the pilot, this could have indicated that they were already requesting specimens from a wider range of patients. We acknowledge that participating practices were interested in the surveillance of AMR and collection of specimens may be slower if sentinel surveillance were implemented on a wider scale. There was no statistically significant difference in the age distribution of patients who submitted specimens to sentinel practices during the pilot compared with previous years and routine data. This casts doubt on whether estimates were based on specimens from a wider range of patients, although we demonstrated that the specimen submission rate during the pilot was increased by 28 specimens per 1000 population per year compared with previous years (95% CI 21–35).

This analysis was conducted prior to the change to the EUCAST susceptibility categories from January 2019, which stipulated that it is no longer appropriate to combine ‘resistant’ and ‘intermediate’ isolates as non-susceptible; however, this would not have affected the conclusions of this study.^21^

### Conclusions and recommendations

Surveillance of AMR in community urinary pathogens based on routine laboratory data resulted in a small overestimation of overall resistance to key antimicrobials, although there were larger differences between sentinel and routine data by age group. Our findings suggest that routine surveillance data provide estimates of resistance for which the absolute biases are not too large, such that decisions about empirical therapy for the management of community acquired UTIs could be based on them. We do not recommend the introduction of large-scale sentinel surveillance. However, a larger study at several sites across England may be warranted to obtain more precise, unbiased estimates of antibiotic non-susceptibility in specific population subgroups and to enable calculation of an adjustment factor that could be applied to estimates based on routine data when considering changes in prescribing policy.

## Supplementary Material

dlaa022_Supplementary_Data
