# Supplementary material for: Routine laboratory surveillance of antimicrobial resistance in community-acquired urinary tract infections adequately informs prescribing policy in England
Source: JAC Antimicrob Resist. 2020 May 27;2(2):dlaa022. doi: 10.1093/jacamr/dlaa022 (PMC8210191; doi:10.1093/jacamr/dlaa022)
Supplement: dlaa022_Supplementary_Data [file dlaa022_supplementary_data.zip › Supplementary_data.docx]

**Supplementary data**

**Table S1****. Catchment population and GP practice characteristics, sentinel GP practices**

| GP Practice | List size | Rural-urban classification | GP Practice catchment deprivation score (IMD 2015)* | Age distribution  (% of registered patients) | | | | | Ethnicity  (% of registered patients) | | | | |
| --- | --- | --- | --- | --- | --- | --- | --- | --- | --- | --- | --- | --- | --- |
|  |  |  |  | 0-14 | 15-44 | 45-64 | 65-74 | 75+ | White | Mixed | Asian | Black | Other non-white |
| A^a^ | 14,734 | Urban | 21.9 | 18.1 | 43.3 | 28.1 | 6.2 | 4.4 | 73.8 | 4.4 | 15.1 | 4.4 | 2.3 |
| B^a^ | 7216 | Urban | 49.1 | 25.3 | 50.7 | 17.5 | 3.2 | 3.3 | 36.2 | 7.0 | 20.4 | 30.0 | 6.4 |
| C^a^ | 21,657 | Urban | 40.5 | 15.1 | 69.2 | 11.9 | 2.2 | 1.6 | 45.1 | 5.7 | 26.0 | 16.8 | 6.4 |
| D^a^ | 3773 | Urban | 48.0 | 24.9 | 48.8 | 19.6 | 3.0 | 3.6 | 37.5 | 7.4 | 19.0 | 30.2 | 5.9 |
| NHS Manchester CCG  GP Practices | 6981^b,c^ | Urban | 40.6 | 19.0^c^ | 51.1 ^c^ | 20.5 ^c^ | 5.3 ^c^ | 4.2 ^c^ | 66.6^d^ | 4.6 ^d^ | 17.1 ^d^ | 8.6 ^d^ | 3.1 ^d^ |

IMD - Index of Multiple Deprivation Score 2015 (<https://www.gov.uk/government/statistics/english-indices-of-deprivation-2015>)

^a^ Data source: PHE Fingertips General Practice Profiles (<https://fingertips.phe.org.uk/profile/general-practice>)

* Lower score indicates lower level of deprivation

^b^ Average list size 2017

^c^ PHE Fingertips General Practice Profile. Population age distribution, NHS Manchester CCG (<https://fingertips.phe.org.uk/profile/general-practice/data#page/9/gid/2000005/pat/152/par/E38000217/ati/7/are/P84068>)

^d^ 2011 Census: Key Statistics for local authorities in England and Wales: Ethnicity (Manchester Unitary Authority) (<https://www.ons.gov.uk/file?uri=/peoplepopulationandcommunity/populationandmigration/populationestimates/datasets/2011censuskeystatisticsforlocalauthoritiesinenglandandwales/r21ewrttableks201ewladv1_tcm77-290595.xls>)
