# Supplementary material for: Routine laboratory surveillance of antimicrobial resistance in community-acquired urinary tract infections adequately informs prescribing policy in England
Source: JAC Antimicrob Resist. 2020 May 27;2(2):dlaa022. doi: 10.1093/jacamr/dlaa022 (PMC8210191; doi:10.1093/jacamr/dlaa022)
Supplement: dlaa022_Supplementary_Data [file dlaa022_supplementary_data.zip › Reviewer report 1.docx]

**Reviewer report 1 (R0 – review of original submission)**

**PLEASE NOTE: JAC-AMR aims to publish your anonymous peer review comments with the final article for transparency. Please indicate in the box below whether you give your consent for us to publish your peer review comments for this article.**

Yes

**Synopsis**

**1. If the article contains original data, does the synopsis adequately reflect the manuscript as a whole, with the most important aspects described (of the introduction, methods and key results, and a balanced conclusion)? If NO, what is required to improve the synopsis? Please use the box below.**

Yes

**Introduction**

**2. Does the introduction provide the necessary background information for readers to understand the article? (e.g. does it describe the problem, current state of knowledge, etc.) If NO, what is required to improve it? Please use the box below**

Yes

**3. Is the research hypothesis and/or aims/objectives of the study clearly stated in the introduction? If NO, what is required to improve it? Please use the box below**

No

**4. Is there superfluous/unnecessary text in the introduction? (Only text that is required for readers to understand why the study has been done should be included). If YES, what text is superfluous and should be removed? Please use the box below**

No

Considering the answers above and any other comments/advice you may have, please indicate what is required to improve the Introduction:

The objectives do not state that resistance estimates were based on E.coli testing results only

**Methods**

**5. Were the methods used suitably robust and appropriate for the research question(s) and/or the aims/objectives of the study? If NO, the article should be rejected, but please advise the authors accordingly in the free-text box below and complete the review in full**

Yes

**6. Are the methods described in enough detail so the reader can understand what was done and interpret the results and conclusions appropriately?**

Yes

**7. Are the methods described in language that a reader who does not specialize in the area of the study/methods used will understand? For example, are the statistical approaches used described in language that non-statistician healthcare professionals will understand? If NO, please indicate what is required to improve the language in the methods section in the box below**

Yes

Considering the above and any other comments/advice you may have, please indicate other aspects of the methods section that could be improved in the free-text box below)

I have serious concerns regarding the representativeness of the study: it is based on a convenience sample of only four urban GP practices in the Manchester area, unavailability of risk factor data did not allow for stratification of patients other than by sex and age-group and antibiotic susceptibility results were only analysed for E. coli. Despite these limitations I would still recommend publication of the pilot study (s. comments in 'General' section below)

**Results**

**8. Given the methods used, are results described accurately, in enough detail and in an appropriate format to adequately inform the discussion section and the authors/readers conclusions? If NO, please indicate what is required to improve the results section in the box below**

Yes

**9. Based on what is described in the methods section, are data/results (that are likely to be available to the authors) missing from the current draft that you think should be presented to improve the manuscript?**

No

**10. Are Tables/Figures clear, required and do they add/complement the results text (they should not simply repeat what is in the text or vice versa)? If NO, please indicate what is required to improve the Tables/Figures in the box below**

Yes

Considering the above and any other comments/advice you may have, please indicate what is required to improve the results section:

see comments Methods section

**Discussion**

**11. Are the discussion and any conclusion(s) reasonable given the stated hypothesis/aims/objectives and results? If NO, please indicate how to improve the discussion in the box below**

Yes

**12. Is there superfluous/unnecessary text in the discussion? (Only text that directly relates to the results and understanding thereof in the context of existing published literature and the limitations of the study should be included.) If YES, please indicate what should be removed to improve the discussion in the box below**

No

**13. Is the Limitations sub-section adequate and reflective of the actual limitations of the study? ? If NO, please indicate how to improve the Limitations section in the box below**

Yes

Considering the above and any other comments/advice you may have, please indicate how to improve the discussion section:

The discussion is well written and reflective of the significant limitations of the study. I would suggest to replace 'offers reassurance' (lines 261 and 333) with 'suggests' and edit the title accordingly to better reflect the uncertainty around the findings

**General**

**14. Is referencing throughout the manuscript satisfactory? (for instance are there statements for which you believe a supporting reference should be cited?) If NO, please indicate how to improve referencing in the box below**

Yes

**15. Is the language used (i.e. spelling, grammar, etc.) of high enough quality to be published as is? If NO, please indicate how to improve the language within the article in the box below**

Yes

**16. Are ethics/other approvals appropriate/adequate and satisfactorily described? ? If NO, please indicate how to improve this in the box below**

Yes

Considering the above and any other comments/advice you may have, please advise the authors how to improve these aspects of the article.

The authors are well aware of the limitations of the pilot study and address/discuss the biases as far as possible which suggests that the current public health policy approach in England to use routine laboratory surveillance data is 'good enough' to inform national prescribing guidance.

Despite the caveats, the study is an important pilot project that should, as the authors state in the Conclusions section, encourage a larger study to obtain more precise, unbiased estimates of antibiotic susceptibility in specific population subgroups.

**Opinion and review articles only**

**17. Please advise the authors in the box below on how to improve their opinion or review article:**

|  **Recommendation** | |
| --- | --- |
|  | Accept (as is with no revisions) |
|  | Minor revision (with anticipation of subsequent acceptance if revisions are adequate) |
|  | Major Revision |
|  | Reject |

| **Would you be willing to review a revision of this manuscript?** | |
| --- | --- |
|  | Yes |
|  | No |

**Comments**

 Comments to the Author

If you have any additional comments to the authors about any aspect of the manuscript not covered in the structured review above, please place these in the box below. If you have no additional comments, please say “No additional comments” in box.

No additional comments
